# Evaluation of quasi-static and dynamic nanomechanical properties of bone-metastatic breast cancer cells using a nanoclay cancer testbed

**DOI:** 10.1038/s41598-021-82664-9

**Published:** 2021-02-04

**Authors:** Sumanta Kar, Dinesh R. Katti, Kalpana S. Katti

**Affiliations:** Center for Engineered Cancer Test Beds, Department of Civil and Environmental Engineering, North Dakota State University, Fargo, ND 58108 USA

**Keywords:** Cancer, Oncology, Materials science, Nanoscience and technology

## Abstract

In recent years, there has been increasing interest in investigating the mechanical properties of individual cells to delineate disease mechanisms. Reorganization of cytoskeleton facilitates the colonization of metastatic breast cancer at bone marrow space, leading to bone metastasis. Here, we report evaluation of mechanical properties of two breast cancer cells with different metastatic ability at the site of bone metastases, using quasi-static and dynamic nanoindentation methods. Our results showed that the significant reduction in elastic modulus along with increased liquid-like behavior of bone metastasized MCF-7 cells was induced by depolymerization and reorganization of F-actin to the adherens junctions, whereas bone metastasized MDA-MB-231 cells showed insignificant changes in elastic modulus and F-actin reorganization over time, compared to their respective as-received counterparts. Taken together, our data demonstrate evolution of breast cancer cell mechanics at bone metastases.

## Introduction

With the advent of novel experimental and modeling methodologies, there has been an increasing interest in investigating the mechanical properties of individual cells to delineate disease mechanisms^1,2^. Increasing evidence supports that altered mechanical properties influence cancer pathogenesis and progression at the cellular level^3^. Several studies have compared the stiffness of cancer cells as compared to their healthy counterparts^4–12^.

The epithelial monolayer, a multicellular system composed of firmly connected adjacent cells via adherens junctions, substantially impacts the progression of many human cancers'^13^. The actin cytoskeleton of healthy epithelial cells is attached to adherens junctions to counteract internal and external mechanical stimuli and bestow mechanical stability^14^. In contrast, cancerous epithelial cells most often exhibit disruption in stable cell–cell adhesion due to alterations in either adherens junctions or actin cytoskeleton^15–17^. A few studies have also compared the distribution/expression of the actin cytoskeleton in breast cancer cells with normal breast cells using fluorescence staining/western blot and observed impaired stress fiber formation/reduced levels of F-actin expression in breast cancer cells as compared to normal breast cells^7,9,18–20^.

Various methods are used to measure mechanical properties of cells, including atomic force microscopy (AFM)^7,8,18,19,21–35^ and direct nanoindentation^36,37^. Intrinsic differences exist between the various mechanobiological experiments conducted in the context of geometry of indentor, its penetration as well as engagement of varying volumes of the cell. AFM based studies are based on cantilever mounted probes that evaluate mechanics of cellular systems based on their approach and retraction of the probes. Several AFM-based reports in the literature report elastic moduli of cancer cells in comparison to their healthy counterparts^6–10,38,39^. A recent AFM study evaluates the important connections between energy metabolism and cell stiffness in breast cancer cells comparing healthy cells and metastatic breast cancer cells^40^ Unique metabolic adaptations of cancer cells in comparison to healthy cells are well known^41^. AFM based microrheology experiments demonstrate use of loss tangent to evaluate malignancy potential of cells^38^. Recent studies also employ the use of AFM in combination with traction force microscopy to evalaute viscoelastic properties as well as and contractile prestress of environment^42^ and development of advanced methodologies with fast force volume and mapping techniques^43^. These newly developed methodologies are highly effective in comparisons of cellular types and characteristics and are likely to have important contributions to further understanding of the cell biology of the numerous cancer types. In the development of in silico approaches of the future, accurate values of mechanical properties as well as bevaviour at metastatic sites are needed. The specific values of elastic moduli of cancer cells reported in several AFM based studies are in the kPa range. There are significant differences in the mechanical probing using AFM based methods and direct nanoindentation. The geometry of the AFM tip that is attached to a cantilever has deflections arising from the flexural and rotational stiffness of the cantilever in addition to the material response to applied load while a vertical penetration enabled with a rigid nanoindentor enables a direct measurement of force and displacement. Direct nanoindentation has been applied to measure static and dynamic properties of several biological materials such as osteoblasts^44–46^, tissue engineered bone nodules^46^, soft and mineralized tissues^47–52^, seashells^53–55^, and dental materials^56,57^. The elastic modulus of cell membrane, and cytoplasm are 1.8 kPa and 0.25 kPa respectively^58,59^ while the various cytoskeletal elements such as actin filaments, intermediate filaments, and microtubulkes have elastic moduli of 1GPa, 1 GPa and 1.9 GPa respectively^60^. The volume fraction of cytoskeleton in eukaryotic cells is reported to be about 16–21% of cell volume^61^. Simplistic calculations estimate cellular moduli in the MPa range based on these compositions and properties of constituents. To this end, our group had previously developed a nanoindentation-based technique as an alternative method for measurements of cellular mechanical properties^36,45^.

Further, changes to the stiffest component of the cell, actin filaments, can influence modulus of the cell and dysregulation in the actin cytoskeleton can lead to softening in cancer cells^3,62–64^. Actin reorganization and polymerization also results in softening of cancer cells^36^. The overall density and 3D-organization of actin have also been reported to be a dominant factor accounting for the changes in the mechanical response of cancer cells^65^.

Loss of cell–cell adhesion and gain of the invasive mesenchymal phenotype are hallmarks of epithelial-mesenchymal transition (EMT) of cancer cells, a process that promotes cancer cells to invade the basement membrane; a physical barrier made up of their adjacent cells^66^. Upregulation of several actin-cytoskeletal-associated proteins, including myosin light chain, α-actinin, integrins, and tropomyosin, has been associated with EMT while reduced expression of pseudopod-enriched proteins including Wiskott-Aldrich syndrome protein (WASP) family members, the actin-related proteins-2/3 (Arp2/3) complex, and cortactin are associated with reversal of EMT or mesenchymal-epithelial transition (MET)^16,67–71^. Moreover, altered expressions of Arp2/3 complex and Wiskott-Aldrich syndrome protein family member 2 (WASP2) have been linked with poor prognosis of breast cancer, indicating a significant role of actin cytoskeleton dynamics in cancer progression^72,73^.Breast cancer is the most prevalent cancer types among women, and it becomes incurable once the disease has metastasized to the bones. Changes in cellular shape and architecture facilitate the colonization of metastatic breast cancer at bone marrow space. Due to the scarcity of accurate and efficient models to replicate cancer progression stages for early detection, most of the patients (80%) with breast cancer metastasized to the bones die within five years^74^. Furthermore, studies done to evaluate the changes in breast cancer cells' mechanical properties during cancer progression at the bone-site are lacking, owing to the lack of suitable models to recapitulate the molecular events. In recent years, three-dimensional (3D) culture systems have attracted substantial attention due to their ability to recapitulate in vivo tumor microenvironment by providing adequate spatial and biophysical cues to mimic molecular events during disease progression as compared to two-dimensional (2D) cellular models. Moreover, 3D culture systems eliminate issues in animal models regarding immunodeficiency, species difference, and uncertain disease pathogenesis^75,76^.

We had earlier reported the development 3D in vitro model for prostate/breast cancer bone metastasis, mimicking MET of breast and prostate cancer in the sequential culture of osteogenically differentiated human mesenchymal stem cells (MSCs) and human prostate/breast cancer cells^77,78^ on nanoclay-based polymer bone mimetic scaffolds^79,80^. The new testbed for bone metastasis also enables evaluation of important signalling pathways during metastasis such as role of Wnt/β-catenin signaling on osteogenesis within the bone microenvironment^81^ and drug resistance^82^. Based on static nanoindentation experiments conducted using the testbed approach of metastasis, we also reported that bone-metastatic prostate cancer cells undergoing MET exhibit significant reduction in stiffness due to F-actin reorganization^36^. Based on these observations, we hypothesized that breast cancer cells grown on 3D bone-mimetic scaffolds would exhibit cell mechanics changes over time due to alterations in actin cytoskeleton dynamics and organization during disease progression. To this end, we evaluated mechanical properties of breast cancer cells grown on 3D bone-mimetic scaffolds using quasi-static and dynamic nanoindentation methods and correlated cell mechanics changes with dysregulation in actin cytoskeleton dynamics using fluorescence staining and mRNA expression of cytoskeleton-related genes.

### Results

## Cancer cells alter cell mechanics in response to 3D culture condition

**Figure 1:**
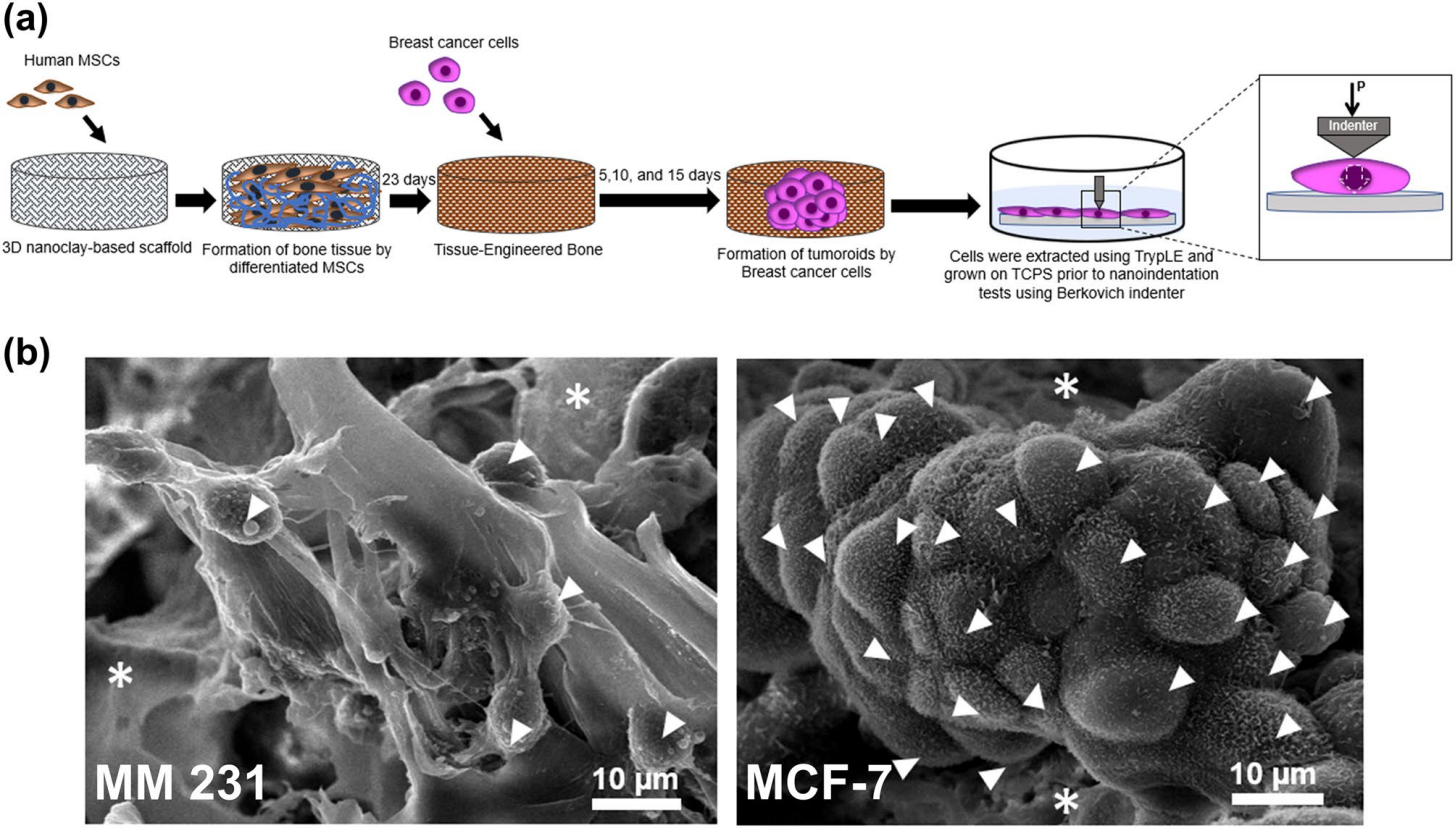
**(a)** Schematic showing steps of sequential culture MSCs/breast cancer followed by workflow of nanoindentation experiment. Dotted arrow pointing downwards indicates the amount of deformation applied (P) onto cell to obtain mechanical response. **(b)** Morphology of breast cancer cells MM 231 and MCF-7 grown on 3D bone-mimetic nanoclay scaffolds. Arrows indicate cells while * indicate scaffold.

According to the procedure described in our previous study, we have created a 3D in vitro model for breast cancer bone metastasis, mimicking MET of breast cancer in the sequential culture of osteogenically differentiated MSCs and breast cancer cells study^77^. Briefly, MSCs were cultured on 3D scaffolds for 23 days to generate bone tissue. Then, human breast cancer cells were seeded on the newly formed bone matrix (Fig. 1a). We observed the formation of tumoroids with distinguishable cellular boundaries by MCF-7 cells, whereas MM 231 cells formed disordered cellular aggregates, as shown in Fig. 1b. Displacement-controlled nanoindentation experiments at maximum displacements of 1000 nm and 2000 nm were performed to obtain mechanical properties of breast cancer cells and elastic moduli were calculated using Oliver-Pharr method^83^. The Oliver-Pharr method takes the unloading load-indentation curve into consideration and therefore is more suitable for capturing a viscoelastic rather than a purely elastic response.

In contrast, other methods such as Hertz and Sneddon lack plastic deformation in the force regime probed for biological materials. Displacement-controlled nanoindentation experiments at maximum displacements of 1000 nm and 2000 nm were performed using TRIBOSCOPE, (Hysitron, Minneapolis, MN) equipped with multimode AFM (NANOSCOPE IIIa controller and J-type piezo scanner system) (Veeco Metrology, Santa Barbara, CA) and a Berkovich diamond indenter fluid tip (three-sided pyramidal; 100–200 nm tip radius). Although earlier studies have used blunt tips for indenting human biological tissue samples^47^, several recent studies have shown that cell stiffness measured by sharp indenters is comparable to stiffness measured by blunt tips^84,85^. Also, sharper tips enable information on the localized mechanical properties from deeper penetration into the probed sample with little to no detrimental effect easily compared to blunt tips^45,85^.

**Figure 2:**
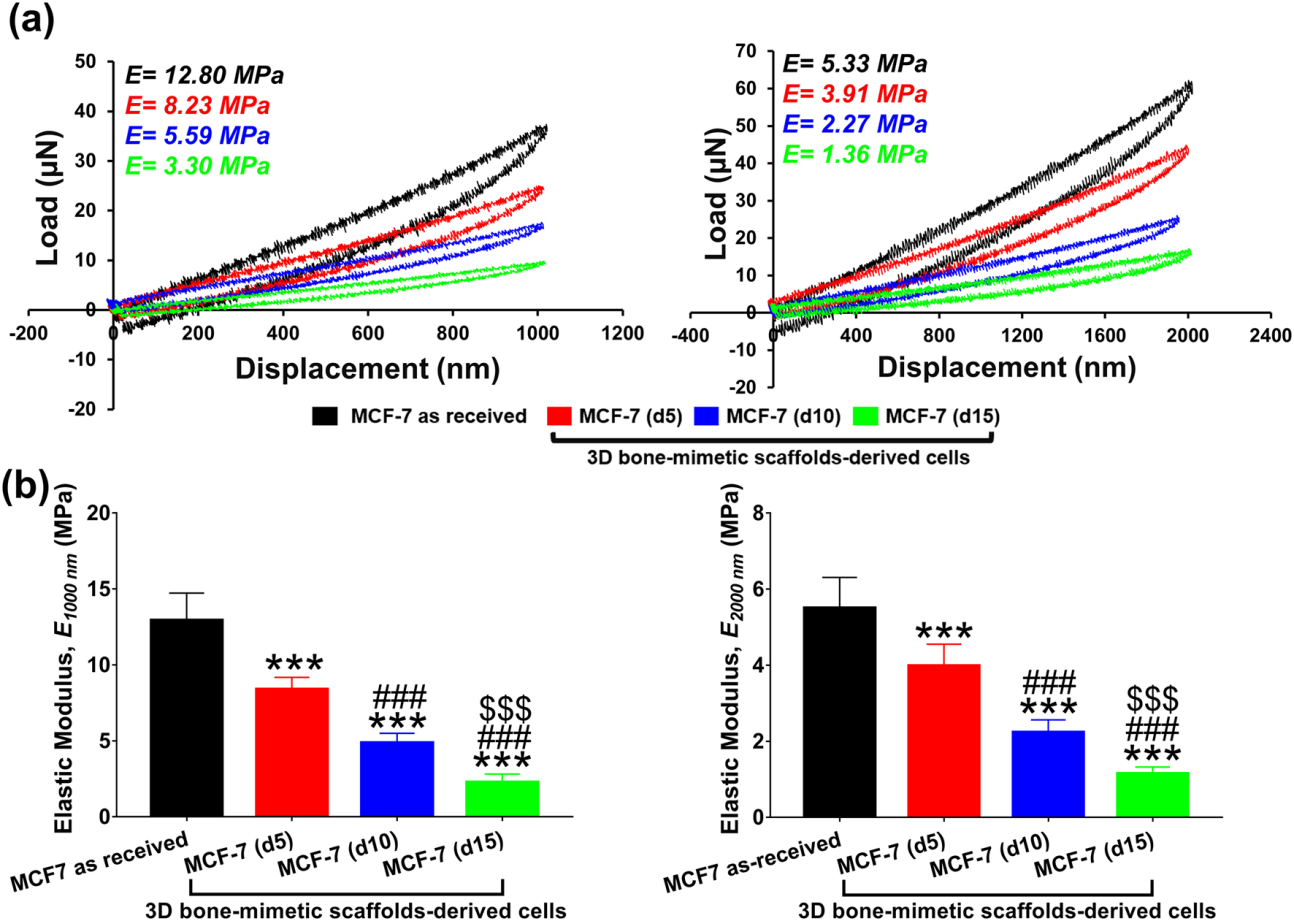
**(a)** Representative load–displacement (L-D) curves of MCF-7 as received, and 3D bone-mimetic scaffolds-derived MCF-7 cells at the maximum depth of 1000 nm and 2000 nm. **(b)** Elastic modulus of MCF-7 as received and 3D bone-mimetic scaffolds-derived MCF-7 cells at the maximum depth of 1000 nm and 2000 nm. For each measured sample, at least 20 cells were measured. Data are reported as a mean ± standard deviation (SD). *p < 0.05, **p < 0.01, and ***p < 0.001 indicate significant difference between MCF-7 as received and 3D bone-mimetic scaffolds-derived MCF-7 cells; ^#^p < 0.05, ^##^p < 0.01, and ^###^p < 0.001 indicate significant difference between scaffolds-derived MCF-7 (d5), and other scaffolds-derived cells (i.e., MCF-7 (d10) and MCF-7 (d15)); ^$^p < 0.05, ^$$^p < 0.01, and ^$$$^p < 0.001 indicate significant difference between scaffolds-derived MCF-7 (d10) and MCF-7 (d15).

Indentation depth has been shown to influence the measurement of the elastic modulus of cells. The mechanical properties observed at ~ 300–500 nm arise from cellular membrane and peripheral structures, whereas, at deeper indentation (~ 1000–2000 nm), the bulk mechanical property of cell can be obtained^85^. The bulk mechanical property of cells arises predominantly from cytoskeletal components such as actin filaments, microtubules, and intermediate filaments^3^. The elastic modulus of each component is given in Table S2. Keeping these facts mentioned above in mind, we used two different indentation depths, 1000 nm and 2000 nm, for displacement controlled nanoindentation experiments. The representative load–displacement (L-D) curves for as-received and 3D bone-mimetic scaffolds-derived MCF-7 cells at the indentation depth of 1000 nm and 2000 nm are shown in Fig. 2a, and the respective elastic moduli are indicated in the figures. Figure 2b shows the variation of elastic modulus between as-received and 3D bone-mimetic scaffolds-derived MCF-7 cells at the maximum indentation depth of 1000 nm and 2000 nm.

We observed that both *E*_*1000 nm*_ and *E*_*2000 nm*_ of scaffolds-derived MCF-7 cells are significantly lower than that of MCF-7 as-received cells. We further observed a significant decrease within the scaffolds-derived MCF-7 cells with an increasing number of days at both 1000 and 2000 nm. It is noteworthy to mention that elastic modulus decreased with increasing indentation depth. At 1000 nm, the mean elastic modulus of MCF-7 as-received, and scaffolds-derived MCF-7 (d5), MCF-7 (d10), and MCF-7 (d15) were 12.92 ± 1.81 MPa, 8.37 ± 0.81 MPa, 4.85 ± 0.65 MPa, and 2.25 ± 0.57 MPa, respectively. At 2000 nm, the mean elastic modulus of MCF-7 as-received, and scaffolds-derived MCF-7 (d5), MCF-7 (d10), and MCF-7 (d15) were 5.50 ± 0.83 MPa, 3.98 ± 0.54 MPa, 2.23 ± 0.32 MPa, and 1.14 ± 0.18 MPa, respectively.

**Figure 3:**
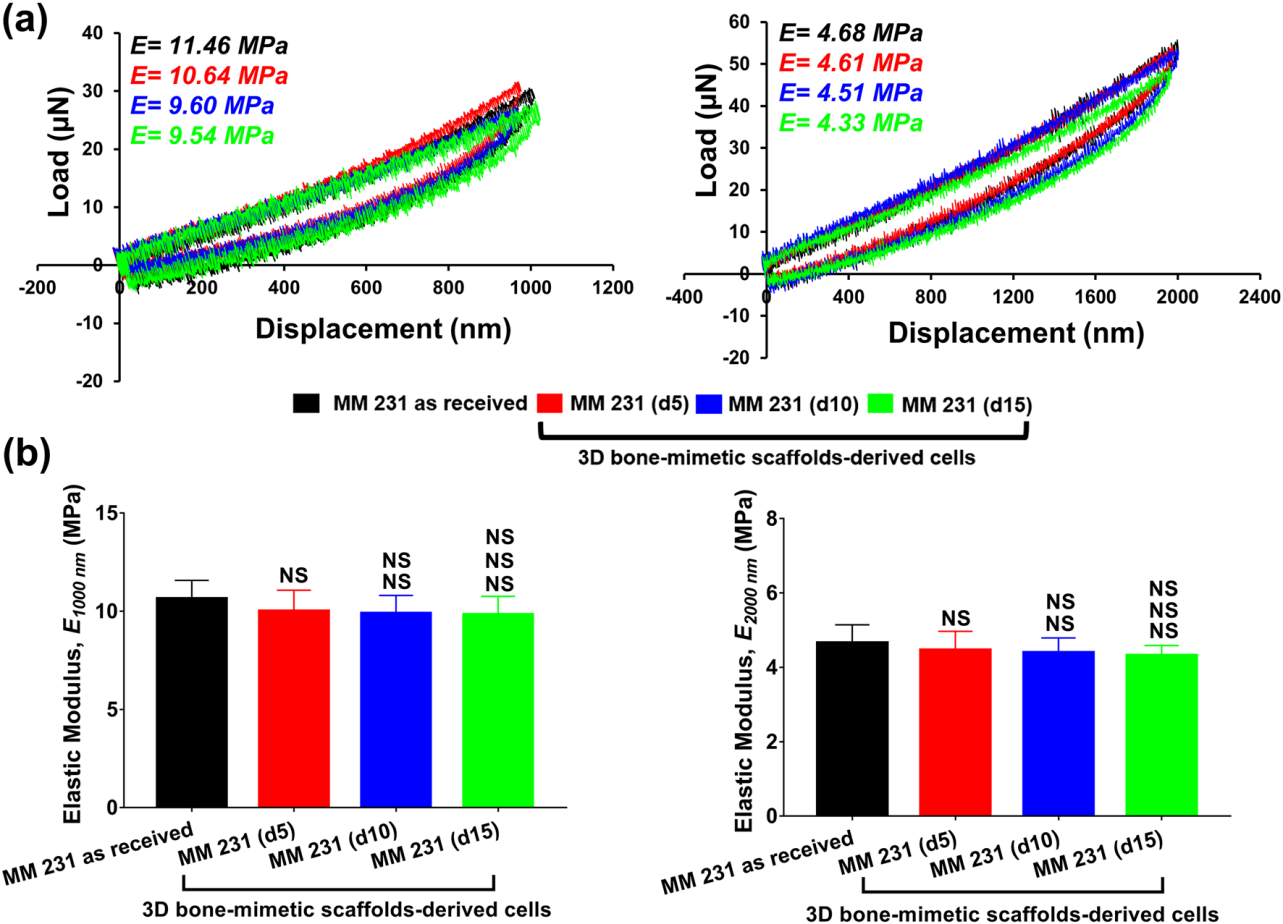
**(a)** Representative load–displacement (L-D) curves of MM 231 as received, and 3D bone-mimetic scaffolds-derived MM 231 cells at the maximum depth of 1000 nm and 2000 nm. **(b)** Elastic modulus of MM 231 as received and 3D bone-mimetic scaffolds-derived MM 231 cells at the maximum depth of 1000 nm and 2000 nm. For each measured sample, at least 20 cells were measured. Data are reported as a mean ± standard deviation (SD). *p < 0.05, **p < 0.01, and ***p < 0.001 indicate significant difference between MM 231 as received and 3D bone-mimetic scaffolds-derived MM 231 cells; ^#^p < 0.05, ^##^p < 0.01, and ^###^p < 0.001 indicate significant difference between scaffolds-derived MM 231 (d5), and other scaffolds-derived cells (i.e., MM 231 (d10) and MM 231 (d15)); ^$^p < 0.05, ^$$^p < 0.01, and ^$$$^p < 0.001 indicate significant difference between scaffolds-derived MM 231 (d10) and MM 231 (d15).

The representative L-D curves for as-received and 3D bone-mimetic scaffolds-derived MM 231 cells at the indentation depth of 1000 nm and 2000 nm are shown in Fig. 3a, and the respective elastic modulus or *E* values are indicated in the figures. Figure 3b shows the elastic modulus of as-received and 3D bone-mimetic scaffolds-derived MM 231 cells at the maximum indentation depth of 1000 nm and 2000 nm. There was no significant difference between elastic moduli of as-received and 3D bone-mimetic scaffolds-derived MM 231 cells at both 1000 nm and 2000 nm. We further observed insignificant differences in elastic modulus of scaffolds-derived MM 231 cells with an increasing number of days at both 1000 and 2000 nm. We once again observed a reduction in elastic modulus with increasing indentation depth. At 1000 nm, the mean elastic modulus of MM 231 as-received, and scaffolds-derived MM 231 (d5), MM 231 (d10), and MM 231 (d15) were 10.62 ± 0.95 MPa, 10.00 ± 1.07 MPa, 9.88 ± 0.93 MPa, and 9.82 ± 0.94 MPa, respectively. At 2000 nm, the mean elastic modulus of MM 231 as-received, and scaffolds-derived MM 231 (d5), MM 231 (d10), and MM 231 (d15) were 4.65 ± 0.49 MPa, 4.46 ± 0.52 MPa, 4.39 ± 0.41 MPa, and 4.31 ± 0.27 MPa, respectively. Overall, MCF-7 showed a progressive reduction in elastic modulus compared to MM 231 when cultured in 3D bone-mimetic scaffolds.

### Cancer cells behave more liquid-like when cultured in 3D culture condition

**Figure 4:**
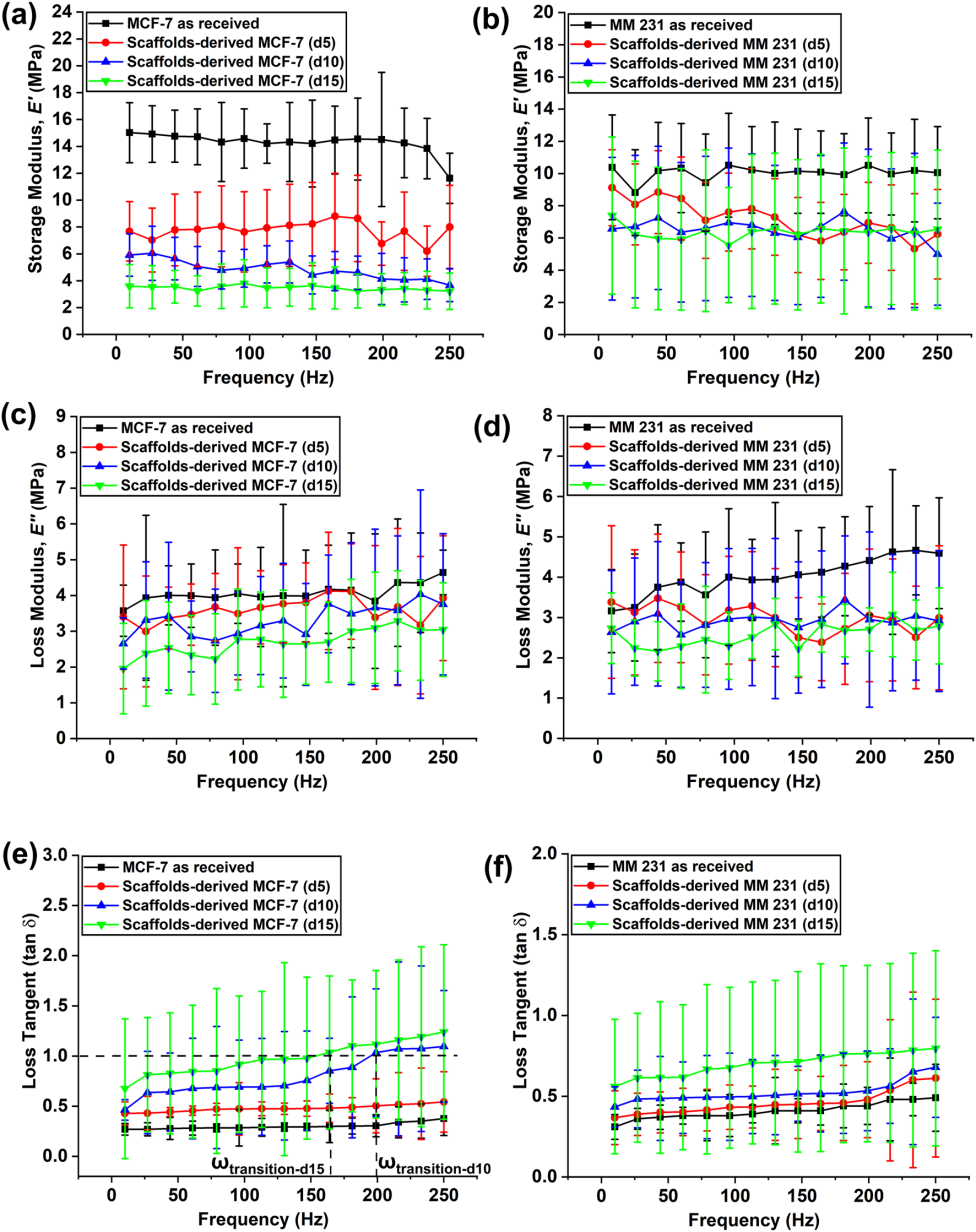
Variation of storage modulus ($E^{\prime}$*)* for **(a)** MCF-7 as received and 3D bone-mimetic scaffolds-derived MCF-7 cells; **(b)** MM 231 as received and 3D bone-mimetic scaffolds-derived MM 231 cells. Variation of loss modulus ($E^{\prime\prime}$*)* for **(c)** MCF-7 as received and 3D bone-mimetic scaffolds-derived MCF-7 cells; **(d)** MM 231 as received and 3D bone-mimetic scaffolds-derived MM 231 cells. Variation of loss tangent (tan δ) for **(e)** MCF-7 as received and 3D bone-mimetic scaffolds-derived MCF-7 cells; **(f)** MM 231 as received and 3D bone-mimetic scaffolds-derived MM 231 cells. Intersections with the horizontal dashed line at tan δ = 1 of the vertical lines occur at transition frequencies, ω_transition_.

The storage modulus ($E^{\prime}$. *)*, loss modulus ($E^{\prime\prime}$*)*, and loss tangent (tan δ) of as-received and scaffolds-derived breast cancer cells (MCF-7 and MM 231) are shown in Fig. 4. Storage moduli of both cells across samples were found to be in the same range as elastic moduli reported in the previous section. For example, storage modulus of MCF-7 as-received, and scaffolds-derived MCF-7 (d5), MCF-7 (d10), and MCF-7 (d15) were ~ 13–14 MPa, ~ 6–8 MPa, ~ 3–6 MPa, and ~ 3–4 MPa, respectively (Fig. 4a). In the case of MM 231, the storage moduli of as-received and scaffolds-derived d5, d10, and d15 we ~ 8–10 MPa, ~ 6–9 MPa, ~ 5–7 MPa, and ~ 5–7 MPa, respectively (Fig. 4b). Loss moduli values for both cells across samples were in the same range. For instance, loss moduli of as-received MCF-7/MM 231, and scaffolds-derived MCF-7/MM 231 (d5), MCF-7/MM 231 (d10), and MCF-7/MM231 (d15) cells were ~ 3–5 MPa, ~ 2–4 MPa, ~ 2–4 MPa, and ~ 2–3 MPa, respectively (Fig. 4c, d). It should be noted that both cells' storage and loss moduli across conditions seem to be independent of frequency. Interestingly, we found an increase in loss tangent (tan δ) with increasing frequency for both cells grown in TCPS (as-received). In other words, cells became more viscous (higher loss tangent, indicating more viscous behavior) at higher frequencies. Next, we assessed whether growing cells on scaffolds altered the viscoelastic properties of the cells. For scaffolds-derived MCF-7 (d5), we noticed an increase in tan δ with increasing frequency, but it never exceeded the value of 1. Interestingly, tan δ values were found to be higher than 1 for both MCF-7 (d10) and MCF-7 (d15) at 199 Hz (and beyond) and 165 Hz (and beyond), respectively. The frequency at which cells transition from solid-like to liquid-like (more viscous) (tan δ = 1) is termed as transition frequency (ω_transition_), as shown by the intersecting dashed lines (Fig. 4e). Based on transition frequency, scaffolds-derived MCF-7 (d15) was found to be more liquid-like as compared to MCF-7 (d10). In the case of scaffolds-derived MM 231 cells, we observed no significant increase in tan δ values over time (Fig. 4f).

We could not determine the transition frequency for MM 231 as tan δ values never went beyond 1. It should be noted that our results are in good agreement with recent studies done on breast cancer cells using high-frequency microrheology based methods^86,87^, and AFM indentation studies^19^. To compare the storage modulus ($E^{\prime}$*)*, loss modulus ($E^{\prime\prime}$*)*, and loss tangent (tan δ) of as-received and bone-site breast cancer cells (MCF-7 and MM 231), we calculated log2 ratios of $E^{\prime}$, $E^{\prime\prime}$, and tan δ for MCF-7 and MM 231 across samples (averaged across frequency) (Figure S1).

We observed a significant decrease in the storage of modulus of scaffolds-derived MCF-7 over scaffolds-derived MM 231 cells over time, as compared to their respective as-received counterparts (Figure S1a). In the case of loss modulus, scaffolds-derived MCF-7 showed a significant increase over scaffolds-derived MM 231 at day 5, compared to their respective as-received counterparts. However, we observed insignificant changes at day 10 and day 15 regarding loss modulus as both cells approached the liquid-like phase during the experiment (Figure S1b). Interestingly, we found a substantial increase in tan δ of scaffolds-derived MCF-7 cells over scaffolds-derived MM 231 over time (Figure S1c), further confirming our observation in Fig. 4e,f. Overall, MCF-7 cells showed more liquid-like behavior than MM 231 cells when cultured in 3D bone-mimetic scaffolds.

### Reorganization of cytoskeleton influences cell mechanics during cancer progression

**Figure 5:**
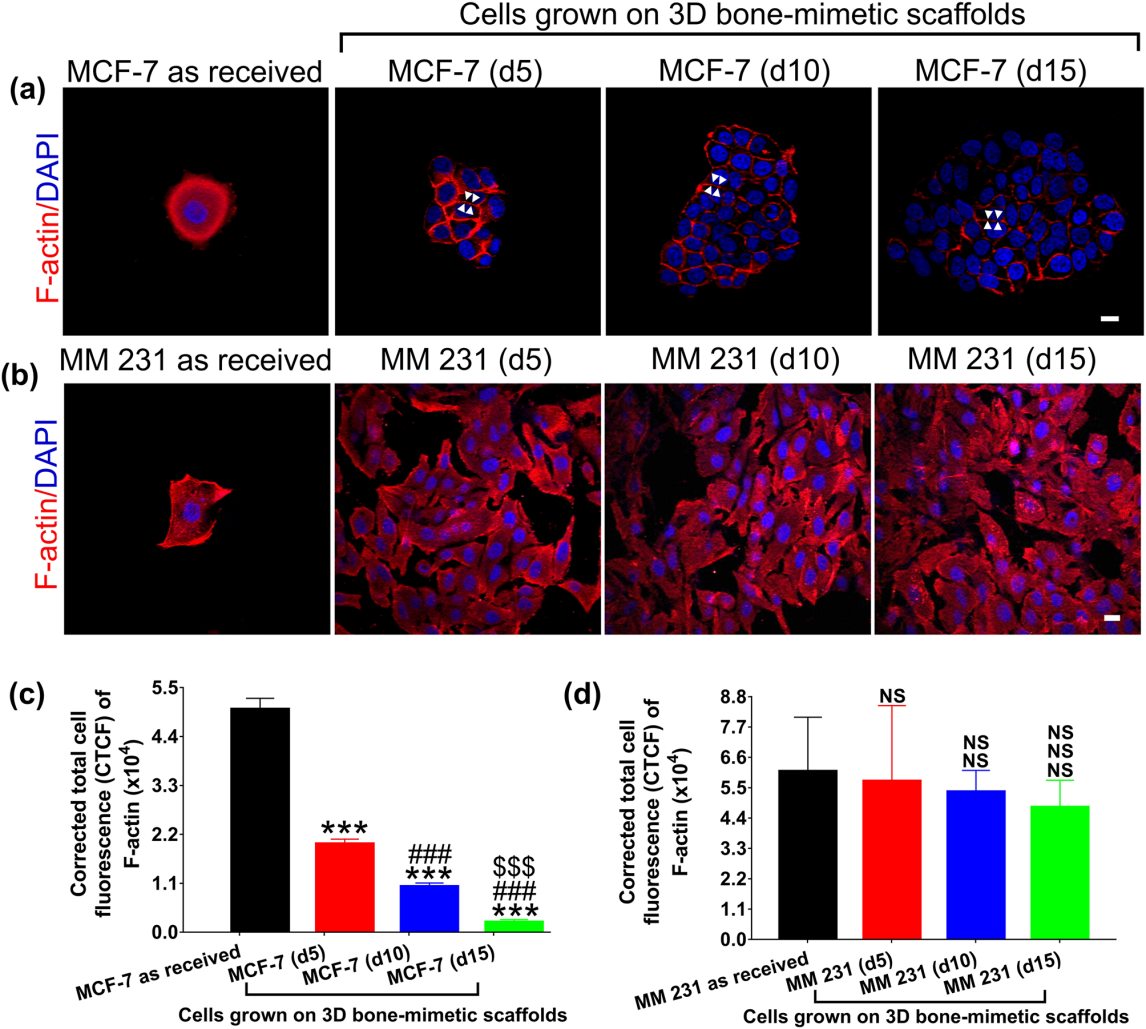
Representative immunofluorescence images showing distribution of F-actin in **(a)** MCF-7 as received and MCF-7 cells grown on 3D bone-mimetic scaffolds. Scaffolds grown MCF-7 formed an F-actin band (shown by white arrows) between adjacent cells; **(b)** MM 231 as received and MM 231 cells grown on 3D bone-mimetic scaffolds. Scaffolds grown MM 231 neither formed an F-actin band between adjacent cells nor stress-fibers. Scale bars: 10 µm. Quantification of corrected total cell fluorescence (CTCF) of F-actin for **(c)** MCF-7 as received and MCF-7 cells grown on 3D bone-mimetic scaffolds; **(d)** MM 231 as received and MM 231 cells grown on 3D bone-mimetic scaffolds. For each measured sample, at least 5–6 cells were measured. Data are reported as a mean ± standard deviation (SD).*p < 0.05, **p < 0.01, and ***p < 0.001 indicate significant difference between as received and 3D bone-mimetic scaffolds-grown breast cancer cells; ^#^p < 0.05, ^##^p < 0.01, and ^###^p < 0.001 indicate significant difference between scaffolds-grown breast cancer cells (d5), and other scaffolds-grown breast cancer cells (i.e., (d10) and (d15)); ^$^p < 0.05, ^$$^p < 0.01, and ^$$$^p < 0.001 indicate significant difference between scaffolds-grown breast cancer cells (d10) and (d15).

To investigate whether the observed changes in elastic moduli and viscoelastic properties were correlated to structural rearrangements of cytoskeletal components, we performed immunofluorescence staining on as-received and scaffolds-grown counterparts of both cancer cells. Representative immunofluorescence images of F-actin and α-tubulin stained as-received MCF-7/MM 231 and MCF-7/MM 231 cells grown on 3D-bone-mimetic scaffolds are shown in Figs. 5 and S2. The immunofluorescence images showed a significant reduction in F-actin intensity in scaffolds-grown MCF-7 cells compared to MCF-7 as-received cells. Still, regardless of culture type, there was no significant difference in the intensity of α-tubulin (Fig. 5a). Furthermore, the F-actin network of as-received MCF-7 consisted of evenly distributed short fibers. MCF-7 cells grown on scaffolds showed thin F-actin band formation between adjacent cells (indicated by small white arrows in Fig. 5a). We calculated the corrected total cell fluorescence (CTCF) of F-actin and α-tubulin for as-received and scaffolds-grown MCF-7 cells to further validate our observation. We found 60.47%, 79.44%, and 95.60% reduction in the intensity of F-actin from as-received to scaffolds-grown MCF-7 (d5), MCF-7 (d10), and MCF-7 (d15), respectively (Fig. 5c); however, we observed no significant changes in the intensity of α-tubulin, as shown in Figure S2a, c. As-received MM 231 cells exhibited an F-actin network consisted of short fibers (Fig. 5b). The organization of the actin cytoskeleton did not change significantly from as-received to scaffolds-grown MM 231 cells. MM 231 cells grown on scaffolds neither formed stress fibers nor an F-actin band between adjacent cells, as shown in Fig. 5b. We once again observed no significant changes in α-tubulin intensity between as-received and scaffolds-grown MM 231 cells (Figure S2b). Furthermore, quantitative analysis of F-actin (Fig. 5d) and α-tubulin fluorescence (Figure S2d) performed on as received and scaffolds-grown MM 231 cells showed good agreement with the observations made in Figs. 5b and S2b, respectively.

**Figure 6:**
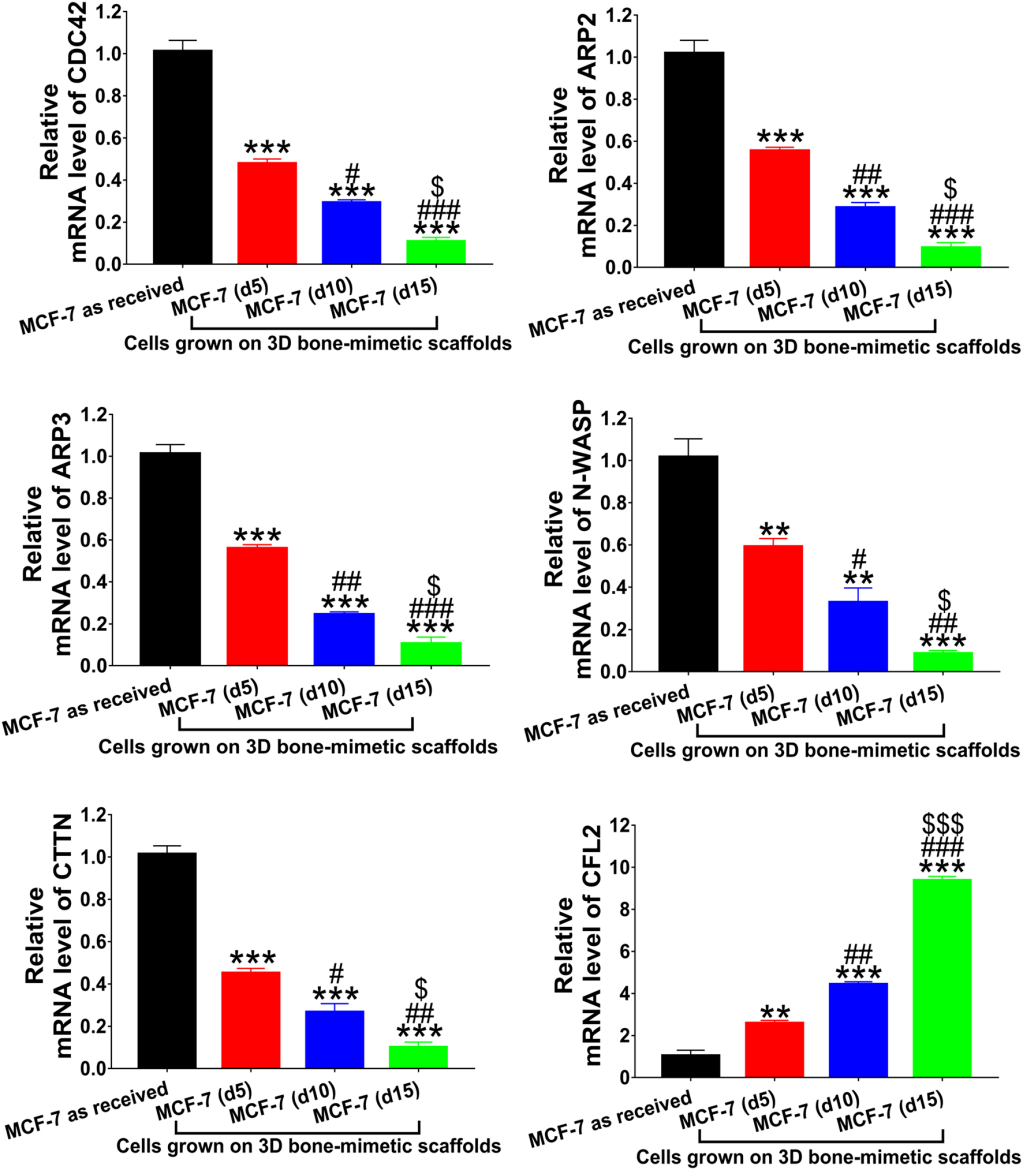
Quantitative real-time PCR of gene expression for actin dynamics-related genes CDC42, ARP2, ARP3, N-WASP, CTTN, and CFL2. *p < 0.05, **p < 0.01, and ***p < 0.001 indicate significant difference between MCF-7 as received and MCF-7 cells grown on 3D bone-mimetic scaffolds; ^#^p < 0.05, ^##^p < 0.01, and ^###^p < 0.001 indicate significant difference between MCF-7 (d5) cells grown on 3D bone-mimetic scaffolds, and other MCF-7 cells grown on 3D bone-mimetic scaffolds (i.e., MCF-7 (d10) and MCF-7 (d15)); ^$^p < 0.05, ^$$^p < 0.01, and ^$$$^p < 0.001 indicate significant difference between MCF-7 (d10) and MCF-7 (d15) cells grown on 3D bone-mimetic scaffolds.

**Figure 7:**
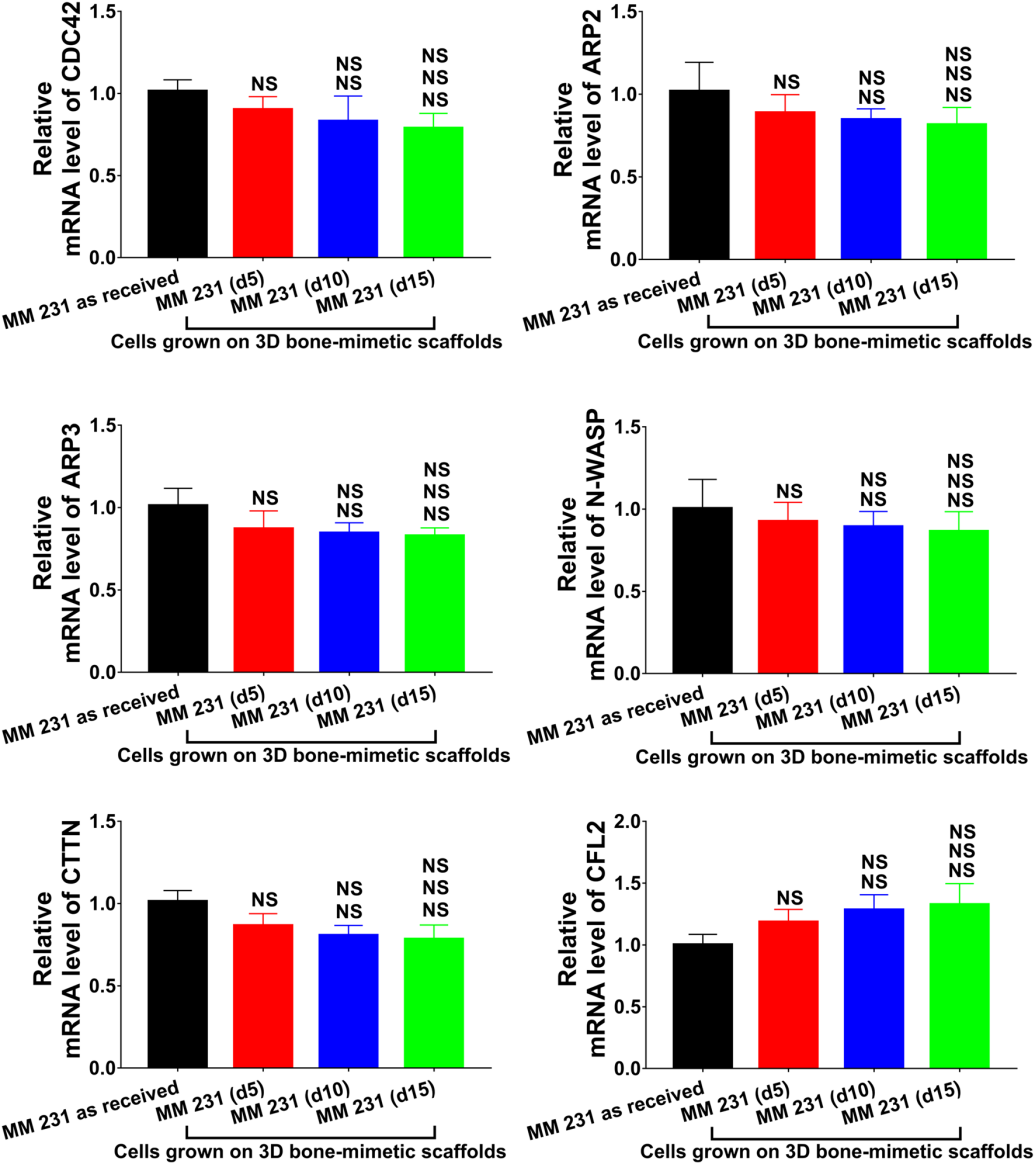
Quantitative real-time PCR of gene expression for actin dynamics-related genes CDC42, ARP2, ARP3, N-WASP, CTTN, and CFL2. *p < 0.05, **p < 0.01, and ***p < 0.001 indicate significant difference between MM 231 as received and MM 231 cells grown on 3D bone-mimetic scaffolds; ^#^p < 0.05, ^##^p < 0.01, and ^###^p < 0.001 indicate significant difference between MM 231 (d5) cells grown on 3D bone-mimetic scaffolds, and other MM 231 cells grown on 3D bone-mimetic scaffolds (i.e., MM 231 (d10) and MM 231 (d15)) ; ^$^p < 0.05, ^$$^p < 0.01, and ^$$$^p < 0.001 indicate significant difference between MM 231 (d10) and MM 231 (d15) cells grown on 3D bone-mimetic scaffolds.

**Figure 8:**
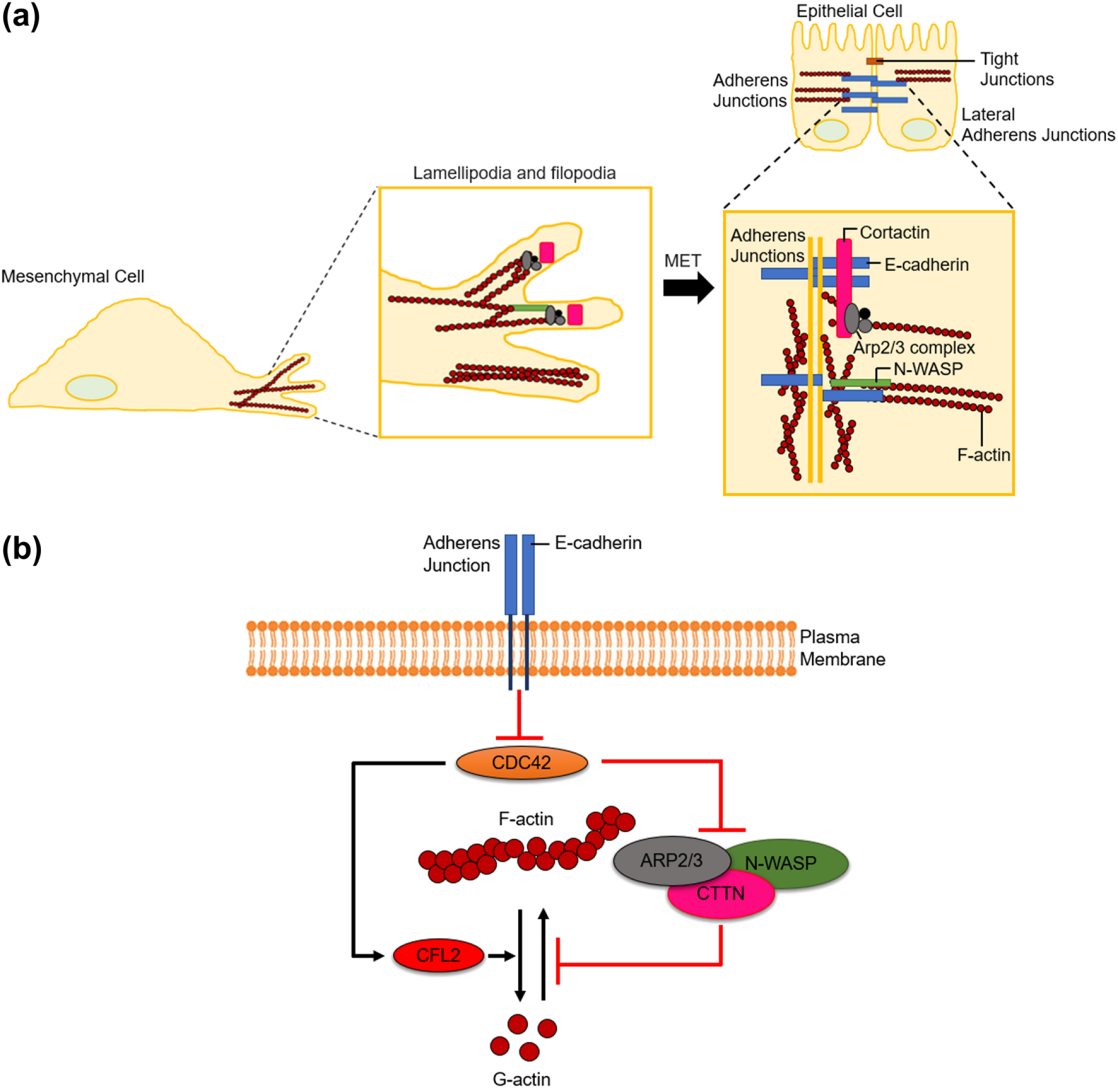
**(a)** In mesenchymal cells, E-cadherin is not present, and the actin cytoskeleton undergoes several changes, leading to a shift of actin and its regulatory proteins and complexes such as N-WASP, cortactin, and Arp2/3 complex from the cortex towards the leading edge to form lamellipodia. In contrast, epithelial cells form tight junctions to create an intracellular barrier separating the tissue from the outside world and adherens junction between adjacent cells that bestow mechanical stability by connecting with the actin cytoskeleton and E-cadherin. Furthermore, E-cadherin maintains adherens junction integrity by providing a basis for sequestration of actin nucleation proteins in non-motile cells. Cortactin, a scaffolding protein, binds to both N-WASP and E-cadherin to recruit Arp2/3 complex to adherens junctions, leading to a reduction in the expression of actin-regulatory proteins. **(b)** E-cadherin inhibits CDC42, a small GTPase of the Rho family, which in turn prevents actin interacting regulatory proteins (N-WASP, ARP2/3, and CTTN) from taking part in actin nucleation, at the same time stimulating the activity of actin severing protein CFL2, thereby promoting depolymerization of actin.

It was previously shown that actin filaments affect the mechanical properties of cells and not microtubules^24,88^. Our results are in good agreement with previous studies. The observations mentioned above prompted us to evaluate the expression of genes related to actin dynamics. We evaluated the expression of CDC42, ARP2, ARP3, N-WASP, CTTN, and CFL2 at the mRNA level. Figure 6 shows the expression of these genes for both as-received and scaffolds-grown MCF-7 cells. CDC42, ARP2, ARP3, N-WASP, and CTTN showed reduction at the mRNA level by ~ 1.70–2.20 fold, ~ 3–4 fold, and ~ 9–12 fold in scaffolds-grown MCF-7 (d5), MCF-7 (d10), and MCF-7 (d15), respectively, compared to as-received MCF-7. Interestingly, we observed an upregulation in CFL2 expression level by ~ 2.58 fold, ~ 4.42 fold, and ~ 9.37 fold in scaffolds-grown MCF-7 (d5), MCF-7 (d10), and MCF-7 (d15), respectively, compared to as-received MCF-7. In the case of MM 231, we observed no significant changes in the expression levels of all the genes evaluated for both as-received and scaffolds-grown cells over time, as shown in Fig. 7. The schematic diagram shown in Fig. 8a shows how actin regulatory proteins mediate the reorganization of the actin cytoskeleton during MET. The mechanism of F-actin depolymerization and reorganization based on our observations is shown in Fig. 8b.

Taken together, these expeiments indicate that actin dynamics seems to regulate cell mechanics during cancer progression, and MCF-7 cells exhibited a substantial reorganization of actin cytoskeleton when cultured in 3D bone-mimetic scaffolds as compared to MM 231 cells.

## Discussion

We investigated the mechanical properties of as-received and 3D bone-mimetic scaffolds-derived breast cancer cells (MCF-7 and MM 231). We found that the bone-site or scaffolds-derived MCF-7 cells exhibit dramatic changes in elastic modulus and demonstrate liquid-like behavior over time compared to MCF-7 as-received cells. In contrast, the bone-site or scaffolds-derived MM 231 cells neither showed significant changes in elastic modulus nor exhibited liquid-like behavior over time than as-received MM 231 cells. Higher loss tangent values of MCF-7 cells compared to MM 231 cells were observed and reported earlier with as-received cells in a study conducted using AFM^19^.

The bone scaffolds-derived MCF-7 cells appear softer and have a larger loss tangent (a measure of liquid-like behavior) than as-received MCF-7 cells (Figs. 2 and 4e). In contrast, elastic moduli and loss tangent values are similar for as-received and scaffolds-derived MM 231 cells (Figs. 3 and 4f). Earlier studies show that dysregulation in actin cytoskeletal dynamics is associated with increased liquidity or liquid-like behavior (represented by higher loss tangent). At the same time, elastic moduli increase with the stabilization of the F-actin network^89^. As received MCF-7 and MDA-MB231 cell lines have been previously investigated for their loss tangents^40^ and more fluid like characteristics of MCF-7 as compared to as recieved MDA-MB-231 cell line is reported. Clearly, on arrival at the bone metastasis location, MCF-7 continues to have increased liquid like behavior while the MDA-MB-231 cells do not exhibit reduction in elastic modulus or liquid-like behavior over time. The inherently higher metastatic potential of MDA-MB-231 cells as compared to the MCF-7 cells manifests as largely different changes to mechanics at the bone site.

Actin cytoskeleton dynamics has been shown to play a critical role during transitions between mesenchymal and epithelial states. E-cadherin is not present in the mesenchymal state, and the actin cytoskeleton undergoes several changes, leading to a shift of actin and its regulatory proteins and complexes such as N-WASP, cortactin, and Arp2/3 complex from the cortex towards the leading edge to form lamellipodia^14^. In contrast, epithelial cells form tight junctions to create an intracellular barrier separating the tissue from the outside world and adherens junction between adjacent cells that bestow mechanical stability by connecting with the actin cytoskeleton and E-cadherin. Furthermore, E-cadherin maintains adherens junction integrity by providing a basis for sequestration of actin nucleation proteins in non-motile cells. Cortactin, a scaffolding protein, binds to both N-WASP and E-cadherin to recruit Arp2/3 complex to adherens junctions^14,71^. Although the Rho family small GTPase CDC42 has been shown to regulate most of the changes in the mesenchymal state^90^, overexpression of E-cadherin (in the epithelial state) has been associated with inhibition of CDC42^69,91^. Thus, the degree of F-actin reduction is closely related to the expression of E-cadherin. We have previously reported an enhanced expression of E-cadherin in bone-site breast cancer cells during MET^77^. This prompted us to calculate the log2 ratios of E-cadherin expression in scaffolds-grown MM 231/MCF-7 compared to their respective controls (Figure S3). We observed a 95.44% increase in E-cadherin expression in scaffolds-grown MCF-7 cells compared to scaffolds-grown MM 231 cells (Figure S3c). Hence, overexpression of E-cadherin inhibits CDC42, a small GTPase of the Rho family, which in turn prevents actin interacting regulatory proteins (N-WASP, ARP2/3, and CTTN) from participating in actin nucleation, at the same time stimulating the activity of actin severing protein CFL2, thereby promoting depolymerization of F-actin (Fig. 8b). This explains the significant reduction of F-actin in scaffolds-grown MCF-7 cells compared to scaffolds-grown MM 231 cells. In summary, we investigated mechanical properties of as received and 3D bone-mimetic scaffolds-derived breast cancer cells (MCF-7 and MM 231). We found that bone-mimetic scaffolds-derived MCF-7 cells exhibited dramatic changes in elastic modulus and demonstrated liquid-like behavior over time compared to MCF-7 as received cells. In contrast, the bone site or scaffolds-derived MM 231 cells neither showed significant differences in elastic modulus nor exhibited liquid-like behavior over time compared to as received MM 231 cells. The bone site or scaffolds-derived MCF-7 cells were softer and had a larger loss tangent (a measure of liquid-like behavior) than as received MCF-7 cells, whereas while elastic moduli and loss tangent values were similar for as received and scaffolds-derived MM 231 cells. From immunofluorescence and gene expression analysis results, we observed that the significant reduction in elastic modulus together with increased liquid-like behavior of scaffolds-derived MCF-7 cells compared to MCF-7 as received cells, was induced by depolymerization and reorganization of F-actin to the adherens junctions. In contrast, scaffolds-grown MM 231 cells showed insignificant changes in F-actin reorganization over time, as opposed to their as received counterparts. The significant reduction of F-actin in MCF-7 cells during progression of metastasis at bone site indicates an increased role of the highly viscoelastic cytoplasm in MCF-7, leading to overall increase in loss tangent. MM-231 cells do not exhibit this behavior. Recent studies also demonstrate the relationship between the cell stiffness and energy metabolism in breast cancer cells showing difference in mechanisms of stiffness in healthy cells as compared to metastatic cells^40^. It is interesting to also note that the pathogenesis of the two cell cells is quite different clinically and here we demonstrate the use of a bone scaffold testbed to create metastasis that can be captured through cell mechanics. Collectively, our results showed evolution of breast cancer cell mechanics at bone metastases.

## Methods

### Materials, cell lines, and cell culture maintenance

Na-MMT clay was procured from Clay Minerals Respiratory at the University of Missouri. Calcium chloride (CaCl_2_), polycaprolactone (PCL) (average Mn 80,000), 1,4-dioxane, sodium phosphate (Na_2_HPO_4_), Tween20, 4′,6-diamidino-2-phenylindole (DAPI), fish skin gelatin (FSG), 5‐aminovaleric acid, and TritonX-100 were purchased from Sigma Aldrich. Human mesenchymal stem cells (MSCs) (PT-2501) were purchased from Lonza (Walkersville, MD) and maintained in complete growth medium (MSCGM SingleQuots (PT‐4105) was added to MSC basal medium (MSCBM, PT‐3238) to obtain complete growth medium). Human breast cancer cell lines MCF-7 (HTB-22), MDA-MB-231 (HTB-26) (shortened as MM 231), Eagle’s Minimum Essential Medium (EMEM), and Fetal Bovine Serum (FBS) were purchased from American Type Culture Collection (ATCC). Corning Phosphate Buffered Saline (PBS), Hyclone Dulbecco’s Modified Eagle medium Nutrient Mixture F-12 DMEM-F-12(1:1), and Alfa Aesar Paraformaldehyde, 4% in PBS (PFA) were purchased from VWR. Rhodamine Phalloidin, Gibco human recombinant insulin, Gibco penicillin–streptomycin antibiotic solution (P/S), Applied Biosystems Fast SYBR Green, and Gibco TrypLE Express Enzyme (1X), phenol red were purchased from Invitrogen. MCF-7 cells were maintained in EMEM, 10% FBS, 0.01 mg/ml human recombinant insulin, and 1% P/S whereas MM 231 cells were cultured in DMEM-F-12(1:1), 10% FBS, and 1% P/S. All cell cultures were maintained at 37 °C and 5% CO_2_ in a humidified incubator. Direct-zol RNA MiniPrep kit (Zymo Research), anti-α-tubulin (Abcam) primary antibody, secondary antibody, and other reagents used were of analytical grade.

### Preparation of PCL/in situ HAPclay 3D scaffolds

PCL/in situ HAPclay scaffolds were synthesized following the procedure described elsewhere^79^. Briefly, we modified clay with 5-aminovaleric acid to increase the d-spacing of clay followed by biomineralization of hydroxyapatite (HAP) into intercalated nanoclay galleries to obtain in situ HAPclay according to the procedure reported in our previous studies^92,93^. Next, 10% in situ HAPclay was added to the PCL solution to get a composite mixture, which was further subjected to freeze-extraction to synthesize PCL/in situ HAPclay scaffolds. Scaffolds were cut into a cylindrical shape (~ 12 mm diameter and ~ 3 mm thickness), sterilized under UV light for 45 min, submerged in 70% ethanol for 12 h, washed in PBS to remove excess ethanol, and stored in a 5% CO_2_ incubator at 37 °C immersed in 24-well plates containing culture medium for 24 h before cell seeding.

### Cell culture

MSCs were seeded at a density of 5 × 10^4^ cells per scaffold and cultured for 23 days to deposit bone-like extracellular matrix (ECM) onto scaffolds. Then, scaffolds with newly formed bone were seeded with 5 × 10^4^ breast cancer cells (MCF-7/ MM231) per scaffold and maintained in 1:1 MSCs and breast cancer cell medium. Breast cancer cells (MCF-7/MM 231) cultured on 2D tissue culture polystyrene (TCPS) are called “as-received” throughout the study.

### Cellular morphology

Cell-seeded scaffold constructs were fixed with 2.5% glutaraldehyde overnight, followed by dehydration in a graded series of ethanol solution (10%, 30%, 50%, 70%, and 100%) and drying in hexamethyldisilazane. Then, samples were gold sputter coated and observed with a JSM-6490LV SEM (JEOL, Tokyo, Japan).

### Quasi-static nanoindentation

The quasi-static mechanical characterization of live breast cancer cells (MCF-7/MM 231) was conducted using TRIBOSCOPE, (Hysitron, Minneapolis, MN) equipped with multimode AFM (Nanoscope IIIa controller and J-type piezo scanner system) (Veeco Metrology, Santa Barbara, CA) and a Berkovich diamond indenter fluid tip (three-sided pyramidal; 100–200 nm tip radius) on displacement-controlled mode at maximum displacements of 1000 nm and 2000 nm corresponding to contact areas of 2.52 × 10^7^ nm^2^ and 9.98 × 10^7^ nm^2^ at a loading and unloading rate of 100 nm/s based on the area function equation for the Berkovich tip. The area function^94^ used for the berkovich tip is

$$A = 24.675h_{c}^{2} + 0.562h_{c} + 0.003216$$

where h_c_ is the penetration in mm and area A is in mm^2^. The breast cancer cells were cultured on TCPS and then glued the TCPS with cells onto a 3D-printed holder (~ 12 mm diameter and ~ 3 mm height) that was filled with fresh culture medium (EMEM + 2% FBS (MCF-7), DMEM-F12 + 2% FBS (MM 231)), which was further glued to a steel disc. The holder containing the cell-seeded TCPS sample was placed onto the nanoindentation stage. The whole assembly was maintained at 37 °C using a MULTIMODE low-temperature heater from Veeco Metrology (Santa Barbara, CA) during the experiment. All the indentation tests were completed within two hours. For cells grown on 3D scaffolds, cells were extracted using TrypLE Express Enzyme and seeded onto TCPS before performing nanoindentation tests. Nanoindentation was done on such extracted tumors and not on individual cells. Damage to the cell resulting in puncture would drastically reduced the cell's elastic modulus because of the collapse of the cell's internal structure. We did not observe this behavior. In addition, if the cell punctures, no recovery of deformation would be observed. The force–displacement curves during unloading show significant recovery indicating that cells have remained intact during the process of loading and unloading. Using the Oliver & Pharr method^83^, the elastic modulus (*E*) of cells was calculated from load–displacement (L-D) curves. In this method, the initial unloading portion of the L-D curve is fitted to power-law function followed by differentiation of power-law relation to obtain contact stiffness. The reduced elastic modulus (*E*_*r*_) of cancer cells was calculated from the stiffness and contact area. Elastic modulus (*E*) of cells was further determined from the reduced modulus (*E*_*r*_), and Poisson’s ratio (*υ*) of 0.50 was used for biological systems using the following equation:

$$\frac{1}{{E_{r} }} = \frac{{\left( {1 - v^{2} } \right)}}{E} + \frac{{\left( {1 - v_{i}^{2} } \right)}}{{E_{i} }}$$

where *υ* and *E* are Poisson’s ratio and the elastic modulus of the sample, respectively; and *υ*_*i*_ and *E*_*i*_ are respective properties of the indenter. For diamond, *υ*_*i*_ = 0.07 and *E*_*i*_ = 1141 GPa. For each measured sample, at least 20 cells were measured. Data are reported as a mean ± standard deviation (SD).

### Dynamic nanoindentation

The dynamic mechanical response of live cancer cells was obtained using frequency sweep mode of nano-DMA module on TRIBOSCOPE, (Hysitron, Minneapolis USA) equipped with multimode AFM (NANOSCOPE IIIa controller and J-type piezo scanner system) (Veeco Metrology, Santa Barbara, CA). In this mode, the oscillating dynamic load of 1 µN was superimposed on a quasi-static load of 1000 µN over a frequency range of 10–250 Hz. The dynamic nanoindentation tests were performed using the same setup and Berkovich diamond indenter, as described in previous section.

In dynamic nanoindentation tests, the displacement amplitude, load amplitude, and phase lag were measured to calculate the storage modulus ($E^{\prime}$*)*, loss modulus ($E^{\prime\prime}$*)*, and loss tangent (tan δ) of cancer cells. During dynamic nanoindentation tests, the sample is subjected to a small oscillatory load (*P*) with a known load amplitude (*P*_*0*_) and frequency (ω). The alternating displacement response is measured at the same testing frequency during the test using a lock-in amplifier. The sinusoidal behavior of the load (*P*) and the resulting displacement (*X*_*0*_) is related to the following expression:

$$\begin{aligned} P & = P_{0} + {\text{sin}}\omega t \\ X & = X_{0} + {\text{sin}}\left( {\omega t{-}\varphi } \right) \\ \end{aligned}$$

where *t* is the time, and φ is the phase difference between load amplitude (*P*_*0*_) and displacement amplitude (*X*_*0*_), respectively. In a dynamic nanoindentation test, the observed response (i.e., the damping coefficient and the stiffness) is the aggregate response of the instrument and the sample being tested. Therefore, the response of the instrument must be subtracted from the aggregate response to obtain the true dynamic properties of the sample. Hence, the stiffness (*k*_*i*_), damping coefficient (*C*_*i*_), and mass (*m*) of the indenter are obtained by air calibration before the experiment, followed by real-time correction of the aggregate response for the response of the instrument. Storage modulus ($E^{\prime}$*)* is given by the in-phase elastic response of the sample, and loss modulus ($E^{\prime\prime}$*)* is a measure of the viscoelastic response of the sample/energy being dissipated during the test. The storage modulus ($E^{\prime}$*)*, loss modulus ($E^{\prime\prime}$*)*, and loss tangent (tan δ) are determined by the following expressions:

$$E^{\prime} = \frac{k\sqrt \pi }{{2\sqrt {A_{c} } }}$$

$$E^{\prime\prime} = \frac{{{\upomega }C\sqrt \pi }}{{2\sqrt {A_{c} } }}$$

$$\tan \delta = \frac{E^{\prime\prime}}{{E^{\prime}}} = \frac{{{\upomega }C}}{k}$$

where *C* and *k* are the damping coefficient and the stiffness of the sample, respectively, and *A*_*c*_ is the projected contact area of indenter on the surface of the sample. For each measured sample, at least 20 cells were measured. Data are reported as mean ± SD.

### Gene expression studies

First, the total RNA was extracted from cell-seeded scaffolds and 2D cultures using the Direct-zol RNA MiniPrep kit followed by reverse-transcription of the extracted RNA to synthesize cDNA using random primers, M‐MLV reverse transcriptase (Promega) on a thermal cycler (Applied Biosystems). Next, we performed real-time polymerase chain reaction was using cDNA, SYBR Green dye, forward primer, reverse primer on a 7500 Fast Real-Time System (Applied Biosystems). The thermal profile used for the run was comprised of a holding stage (2 min at 50 °C, 10 min at 95 °C) and a cycling stage (40 cycles of 15 s at 95 °C, and 1 min at 60 °C). The mRNA expression of Neural-Wiskott-Aldrich syndrome protein (N-WASP), cell division control protein 42 homolog (CDC42), ARP2, ARP3, cortactin (CTTN), E-cadherin (CDH1), and cofilin-2 (CFL2) were quantified and normalized to housekeeping gene glyceraldehyde-3-phosphate-dehydrogenase (GAPDH). The relative expression of mRNAs was determined using the comparative C_t_ method (2^−ΔΔCt^). Table S1 lists the sequence of primers used. All the experiments were repeated on triplicate samples.

### Immunofluorescence staining

Cells grown on scaffolds and 2D cultures were fixed with paraformaldehyde, 4% in PBS for 30 min, permeabilized with TritonX-100, 0.2% in PBS for 5 min, and blocked with FSG, 0.2% in PBS for 45 min. The samples were then incubated overnight at 4 °C with primary antibody diluted in a blocking buffer (0.2% FSG in PBS containing 0.02% Tween20) at a dilution of 1:150. Next, the samples were incubated with Alexa Flour 488 conjugated secondary antibody corresponding to the origin of the primary antibody used for 45 min at 25 °C. The actin cytoskeleton and Nuclei were stained with Rhodamine Phalloidin and DAPI, respectively. The stained samples were observed under Zeiss AxioObserver.Z1 LSM 700. Images were processed with ImageJ software and the quantitative analysis of both F-actin and α-tubulin fluorescence was performed on at least 5–6 cells for each sample evaluated by calculating the corrected total cell fluorescence (CTCF) of each signal as follows:

CTCF = Integrated density of selected cell—(Area of selected cell × Mean fluorescence of background).

### Statistical analysis

Data are reported as mean ± SD. Statistical significance (p-value) among multiple comparisons was determined using one-way ANOVA followed by the post hoc Tukey test, whereas Student’s unpaired *t-test* was used to determine statistical significance (p-value) between two groups, using GraphPad Prism v7.04. *p* < *0.05* was considered statistically significant.

## Supplementary Information

Supplementary Information
