# Supplementary material for: Evaluation of quasi-static and dynamic nanomechanical properties of bone-metastatic breast cancer cells using a nanoclay cancer testbed
Source: Sci Rep. 2021 Feb 4;11:3096. doi: 10.1038/s41598-021-82664-9 (PMC7862348; doi:10.1038/s41598-021-82664-9)
Supplement: Supplementary file 1 — Supplementary Information [file 41598_2021_82664_MOESM1_ESM.docx]

**Supporting Information**

**Evaluation of quasi-static and dynamic nanomechanical properties of bone-metastatic breast cancer cells using a nanoclay cancer testbed**

Sumanta Kar, Dinesh R. Katti, and Kalpana S. Katti*,

Center for Engineered Cancer Test Beds, Materials and Nanotechnology Program, & Department of Civil and Environmental Engineering

North Dakota State University, Fargo ND 58108, USA

* Corresponding Author

Kalpana.katti@ndsu.edu, Ph: 701-231-9504


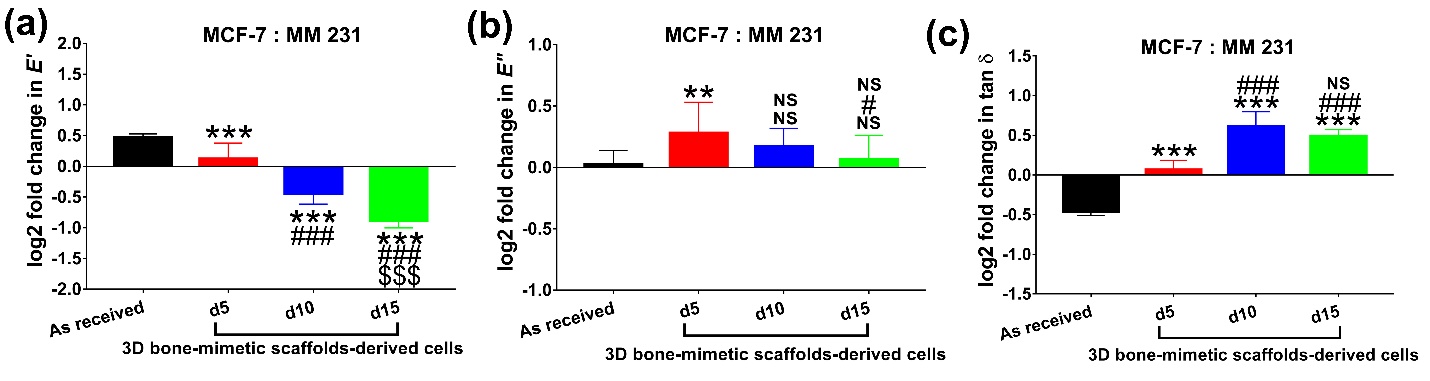


**Fig. S1.** Log2 ratios of **(a)** storage modulus ($E'$*)*, **(b)** loss modulus ($E''$*)*, and **(c)** loss tangent (tan δ) for MCF-7 and MM 231 across samples (mean ± SD, averaged across frequency). *p<0.05, **p<0.01, and ***p<0.001 indicate significant difference between as received and 3D bone-mimetic scaffolds-derived breast cancer cells; ^#^p<0.05, ^##^p<0.01, and ^###^p<0.001 indicate significant difference between scaffolds-derived breast cancer cells (d5), and other scaffolds-derived breast cancer cells (i.e., (d10) and (d15)); ^$^p<0.05, ^$$^p<0.01, and ^$$$^p<0.001 indicate significant difference between scaffolds-derived breast cancer cells (d10) and (d15).


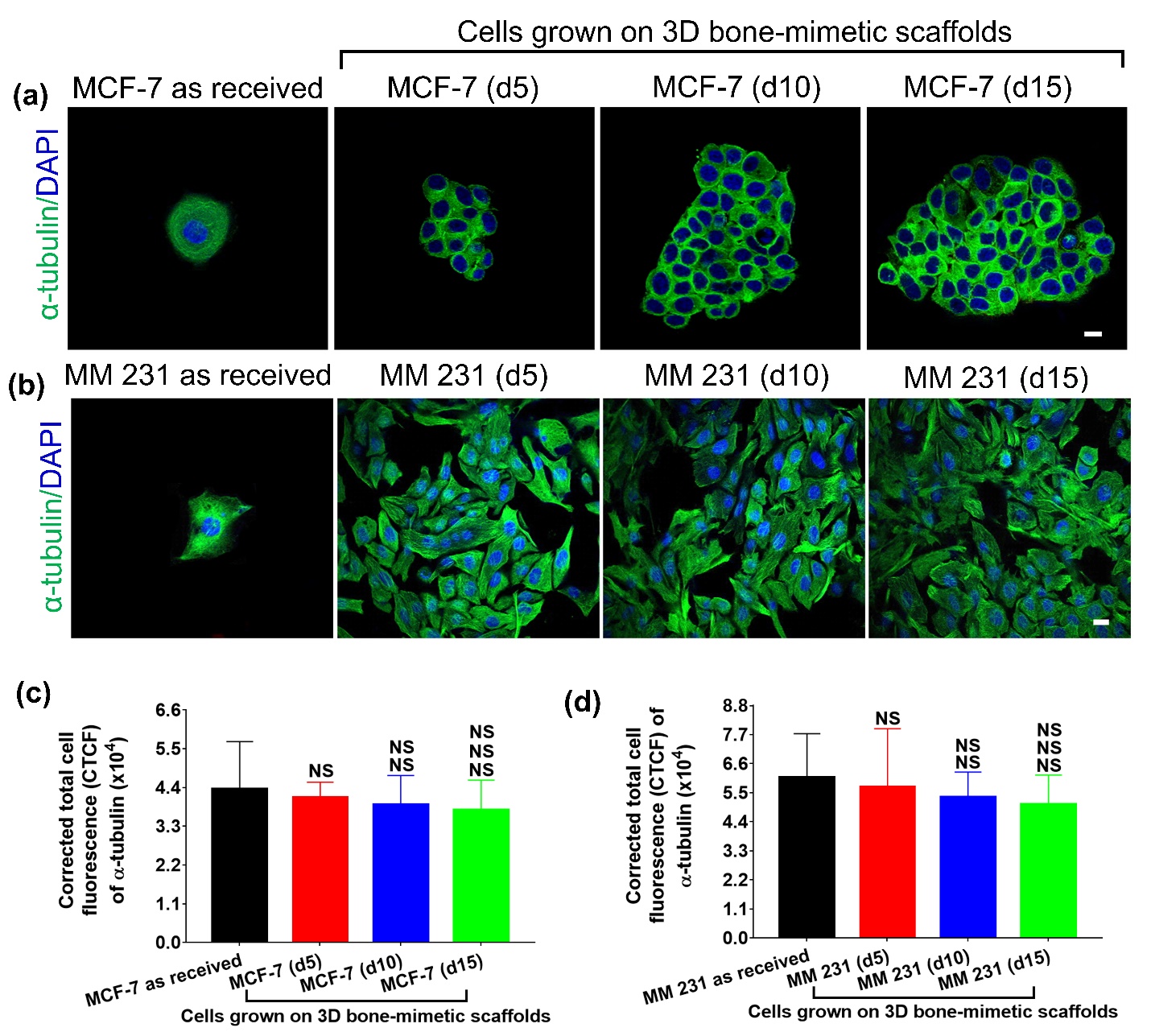


**Fig. S2.** Representative immunofluorescence images showing distribution of α-tubulin in **(a)** MCF-7 as received and MCF-7 cells grown on 3D bone-mimetic scaffolds; **(b)** MM 231 as received and MM 231 cells grown on 3D bone-mimetic scaffolds. Scale bars: 10 µm. Quantification of corrected total cell fluorescence (CTCF) of α-tubulin for **(c)** MCF-7 as received and MCF-7 cells grown on 3D bone-mimetic scaffolds; **(d)** MM 231 as received and MM 231 cells grown on 3D bone-mimetic scaffolds. For each measured sample, at least 5-6 cells were measured. Data are reported as a mean ± standard deviation (SD).*p<0.05, **p<0.01, and ***p<0.001 indicate significant difference between as received and 3D bone-mimetic scaffolds-grown breast cancer cells; ^#^p<0.05, ^##^p<0.01, and ^###^p<0.001 indicate significant difference between scaffolds-grown breast cancer cells (d5), and other scaffolds-grown breast cancer cells (i.e., (d10) and (d15)); ^$^p<0.05, ^$$^p<0.01, and ^$$$^p<0.001 indicate significant difference between scaffolds-grown breast cancer cells (d10) and (d15).


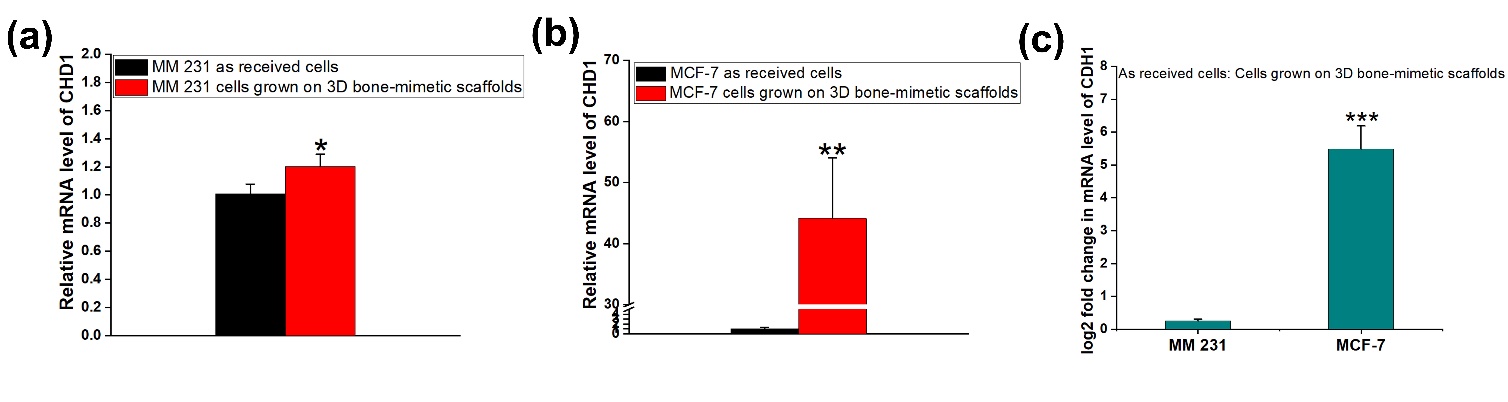


**Fig. S3.** **(a & b)** Quantitative real-time PCR of gene expression for CHD1 for as received MCF-7/MM 231 and MCF-7/MM 231 cells grown 3D bone-mimetic scaffolds. *p<0.05, **p<0.01 indicate significant difference between as received (MM 231/MCF-7) breast cancer cells and cells (MM 231/MCF-7) grown on 3D bone-mimetic scaffolds, **(c)** Log2 ratios of CHD1 gene expression of as received and cells grown on 3D bone-mimetic scaffolds for both breast cancer cells. ***p<0.001 indicates significant difference between MM 231 and MCF-7.


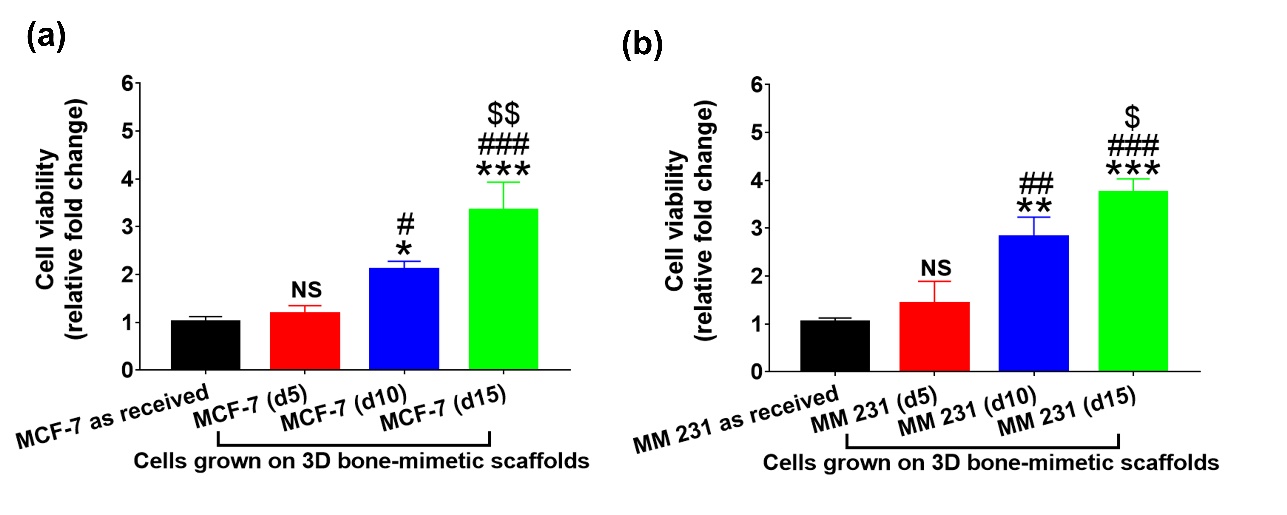


**Fig. S4.** **(a & b)** Cell viability of breast cancer cells (MCF-7/MM 231) grown on 3D bone-mimetic scaffolds over time is demonstrated as fold change in viability from as received (MCF-7/MM 231) counterparts. *p<0.05, **p<0.01, and ***p<0.001 indicate significant difference between as received and 3D bone-mimetic scaffolds-grown breast cancer cells; ^#^p<0.05, ^##^p<0.01, and ^###^p<0.001 indicate significant difference between scaffolds-grown breast cancer cells (d5), and other scaffolds-grown breast cancer cells (i.e., (d10) and (d15)); ^$^p<0.05, ^$$^p<0.01, and ^$$$^p<0.001 indicate significant difference between scaffolds-grown breast cancer cells (d10) and (d15). Cell viability was determined using WST-1 (Roche) as per the manufacturer’s protocol. Briefly, cell-seeded scaffolds and as received samples were washed with PBS, and incubated with a solution consisting of 450 µl DMEM-F-12 (1:1) and 50 µl WST-1 reagent per well for 4 h in a humidified incubator with 5% CO_2_ incubator at 37 ºC. Then, absorbance was measured at 450 nm using a microplate reader.

**Table S1.** The sequence of primers used for the quantitative real-time PCR experiment

| Gene | Forward primer | Reverse primer |
| --- | --- | --- |
| GAPDH | 5’‐CATCTTCTTTTGCGTCGCCA‐3’ | 5’‐TTAAAAGCAGCCCTGGTGACC‐3’ |
| N-WASP | 5’ -ACTGTTAGACCAGATACG ACAGGGT-3’ | 5’-TGCAGGTGTTGGTGGTGT AGA-3’ |
| ARP2 | 5’-GGAGTTGGTGTTGCTGAAT-3’ | 5’-TAGTAGACCCTCCAGAAAGC-3’ |
| ARP3 | 5’-CAATCCTTGGAAACTGCTA-3’ | 5’-CCATTTTGACCCATCTGTA-3’ |
| CDC42 | 5’-ATGCAGACAATTAAGTGTG TTGTTGTGGGCGA-3’ | 5’- TCATAGCAGCACACACC TGCGGCTCTTCTT-3’ |
| CTTN | 5’-AAAGCTTCAGCAGGCCAC-3’ | 5’-TTTGGTCCTGTTTCAAGT TCC-3’ |
| CFL2 | 5’-AGTGCCACAGAGCCGAAG-3’ | 5’-TCATCATTCACTGTAACTCCAGAAG-3’ |
| CDH1 | 5’‐AAG TGA CCG ATGATGAT‐3’ | 5’‐CTC TGT CCA TCT CAG CG‐3’ |

| Cellular component | Elastic modulus |
| --- | --- |
| Actin filaments  Intermediate filaments  Microtubules  Cytoplasm  Cell membrane | 1 GPa  1 GPa  1.9 GPa  0.25 kPa  1.8 kPa |

**Table S2.** Elastic modulus of cellular components ^1-3^

1 Barreto, S., Clausen, C. H., Perrault, C. M., Fletcher, D. A. & Lacroix, D. A multi-structural single cell model of force-induced interactions of cytoskeletal components. *Biomaterials* **34**, 6119-6126 (2013).

2 Suresh, S. Biomechanics and biophysics of cancer cells. *Acta biomaterialia* **3**, 413-438 (2007).

3 Yokokawa, M., Takeyasu, K. & Yoshimura, S. H. Mechanical properties of plasma membrane and nuclear envelope measured by scanning probe microscope. *Journal of microscopy* **232**, 82-90 (2008).

**References**
